# An assessment of Ebola-related stigma and its association with informal healthcare utilisation among Ebola survivors in Sierra Leone: a cross-sectional study

**DOI:** 10.1186/s12889-020-8279-7

**Published:** 2020-02-05

**Authors:** Peter Bai James, Jonathan Wardle, Amie Steel, Jon Adams

**Affiliations:** 1 Australian Research Centre in Complementary and Integrative Medicine, Faculty of Health, University of Technology Sydney, Ultimo, Sydney, NSW 2007 Australia; 2 Faculty of Pharmaceutical Sciences, College of Medicine and Allied Health Sciences, University of Sierra Leone, Freetown, Sierra Leone

**Keywords:** Stigma, Ebola, Ebola survivor, Informal health care, Traditional medicine, Sierra Leone

## Abstract

**Background:**

We examined the magnitude and correlates of Ebola virus disease (EVD)-related stigma among EVD survivors in Sierra Leone since their return to their communities. In addition, we determined whether EVD-related stigma is a predictor of informal health care use among EVD survivors.

**Methods:**

We conducted a cross-sectional study among 358 EVD survivors in five districts across all four geographic regions (Western Area, Northern Province, Eastern Province and Southern Province) of Sierra Leone. Ebola-related stigma was measured by adapting the validated HIV related stigma for people living with HIV/AIDS instrument. We also measured traditional and complementary medicine (T&CM) use (as a measure of informal healthcare use). Data were analysed using descriptive statistics and regression analysis.

**Results:**

EVD survivors report higher levels of internalised stigma (0.92 ± 0.77) compared to total enacted stigma (0.71 ± 0.61). Social isolation (0.96 ± 0.88) was the highest reported enacted stigma subscale. Ebola survivors who identified as Christians [AOR = 2.51, 95%CI: 1.15–5.49, *p* = 0.021], who perceived their health to be fair/poor [AOR = 2.58, 95%CI: 1.39–4.77. *p* = 0.003] and who reside in the northern region of Sierra Leone [AOR = 2.80, 95%CI: 1.29–6.07, *p* = 0.009] were more likely to experience internalised stigma. Verbal abuse [AOR = 1.95, 95%CI: 1.09–3.49, *p* = 0.025] and healthcare neglect [AOR = 2.35, 95%CI: 1.37–4.02, *p* = 0.002] were independent predictors of T&CM use among EVD survivors.

**Conclusion:**

Our findings suggest EVD-related stigma (internalised and enacted) is prevalent among EVD survivors since their return to their communities. Religiosity, perceived health status and region were identified as independent predictors of internalised stigma. Verbal abuse and healthcare neglect predict informal healthcare use. EVD survivor-centred and community-driven anti-stigma programs are needed to promote EVD survivors’ recovery and community re-integration.

## Background

The 2014–2016 Ebola virus disease (EVD) outbreak in West Africa is considered the largest and unprecedented public health emergency in the history of the disease [1]. As at the 30th March 2016, the morbidity and mortality figures due to EVD were estimated at 28, 646 and 11, 322 respectively [2]. The West African Ebola outbreak also recorded the highest number of survivors, and current estimates put the number of EVD survivors at more than 10,000 [3]. Many EVD survivors are known to be suffering from short and long-term physical symptoms and mental complications as a result of surviving EVD [4–6]. Psychosocial consequences of EVD survivorship can be traumatic, due to the adverse psychological experiences of individuals with EVD had to grapple with during infection, treatment and post-discharge. These adverse experiences includes various forms of psychosocial challenges such as depression, anxiety and grief due to loss of loved ones and stigma [4].

Stigma constitutes negative attitudes and beliefs that discredit an individual or group of individuals leading to prejudice and societal exclusion [7]. Stigma can lead to experiences and feelings of blame, shame, worthlessness, loneliness, isolation, social exclusion and discrimination in accessing social amenities and healthcare services [8, 9]. Socially undesirable manifestations (prejudice and discrimination) expressed against those with the stigmatizing attributes are known as enacted stigma whereas the feeling of shame, guilt or worthlessness experienced as a result of having the stigmatising attribute is referred to as internalised stigma [10]. EVD-related stigma is largely based on community fear that EVD survivors are still contagious [11]. Such fear is profound if EVD survivors experience post-Ebola sequelae [12, 13] or are aware that the Ebola virus can be present in certain immune-protective parts of the body after convalescence (for example, the semen, breast milk, ocular (eye) fluid, and spinal column fluid) [14, 15]. EVD-related stigma has led to EVD survivors being mocked by their communities [16, 17], being evicted from their homes by their property owners [13, 17], losing their former jobs [11] and being divorced by their spouses [12, 13]. Some EVD survivors have been prevented from visiting public places such as public toilets and have experienced difficulty in trading commodities at their local market due to a community reluctance to touch their items or money [12, 13]. EVD-related stigma has been reported by EVD survivors and their communities in DR Congo (35%), Guinea (26%) and Liberia (3%) [18–20], and may be more common among female rather than male EVD survivors [12]. Other factors, which have been reported as predictors of EVD-related stigma, are age, level of education, and having accessed medical care [21]. Liberian research also suggests EVD survivors are reported to be more likely to experience stigma compared to their close contacts who were not infected with EVD virus [22] however the degree of EVD-related stigma may decline among survivors over time [21, 23]. In Sierra Leone, stigmatisation is reported in approximately one third of EVD survivors [24, 25].

Stigma associated with infectious disease has been linked to poor adherence to conventional treatment and the utilization of informal or non-integrated forms of health care such as traditional and complementary medicine (T&CM) [26, 27]. T&CM refers to a number of health systems, products and practices considered to be predominantly outside conventional medical practice and the medical curriculum [28, 29]. In sub-Saharan Africa, an average of 58% of the general population is estimated to use T&CM products and 29% consult T&CM practitioners [30]. The key reasons for T&CM use in Africa have been attributed to its low cost, easy accessibility, the alignment between T&CM philosophy and local cultural and religious values, perceived safety and efficacy, and dissatisfaction with conventional medicine [30]. In Sierra Leone, T&CM utilisation is common especially among hypertensive, pregnant women, infertile women, and lactating mothers and in the management of malaria and diarrhoea [31–36]. Studies have reported individuals with HIV/AIDS or mental health diagnoses that experience stigma are more likely to access T&CM services [37, 38]. This pattern of use is reportedly due to the users’ perception of T&CM as less stigmatizing than conventional medicine, partly justified by the view that these T&CM approaches are deeply rooted in the local cultural and traditional practices [37, 38]. Among SARS survivors, T&CM was reported to be useful in overcoming SARS-related stigmas by creating new social support networks and counteracting potential future stigmatization and discrimination [39].

Most studies on stigma among EVD survivors have focussed on its magnitude and nature both immediately following and over a number of years after discharge from an Ebola treatment centre [11–13, 17–21, 23, 25, 40]. Although recent studies have reported the use of informal healthcare services among EVD survivors [41, 42], globally, no study to date has reported whether EVD-related stigma is associated with T&CM utilisation among EVD survivors. In addition, none of the published studies in Sierra Leone on EVD survivors has explored the sociodemographic and health-related factors associated with EVD-related stigma. Such associations are important, as they will inform the design and implementation of future anti-stigma interventions. Therefore, we examined the magnitude and the sociodemographic and health related correlates of enacted and internalised stigma among EVD survivors in Sierra Leone since their return to their communities. In addition, our study determined whether enacted and internalised stigma are possible predictors of informal healthcare service utilisation (T&CM use) among EVD survivors in Sierra Leone.

## Methods

### Study design, setting and participants

We conducted a cross-sectional questionnaire study between January and August 2018 among EVD survivors across all four geographic regions (Western Area, Northern Province, Eastern Province and Southern Province) of Sierra Leone. Participants in this study were adult EVD survivors aged 18 years and older experiencing post-Ebola sequelae. We excluded EVD survivors whose physical and psychological health limited them from providing information, such as those survivors with memory loss, hearing loss, high fever and bleeding or those experiencing acute emotional distress.

### Sampling method

**Fig. 1:**
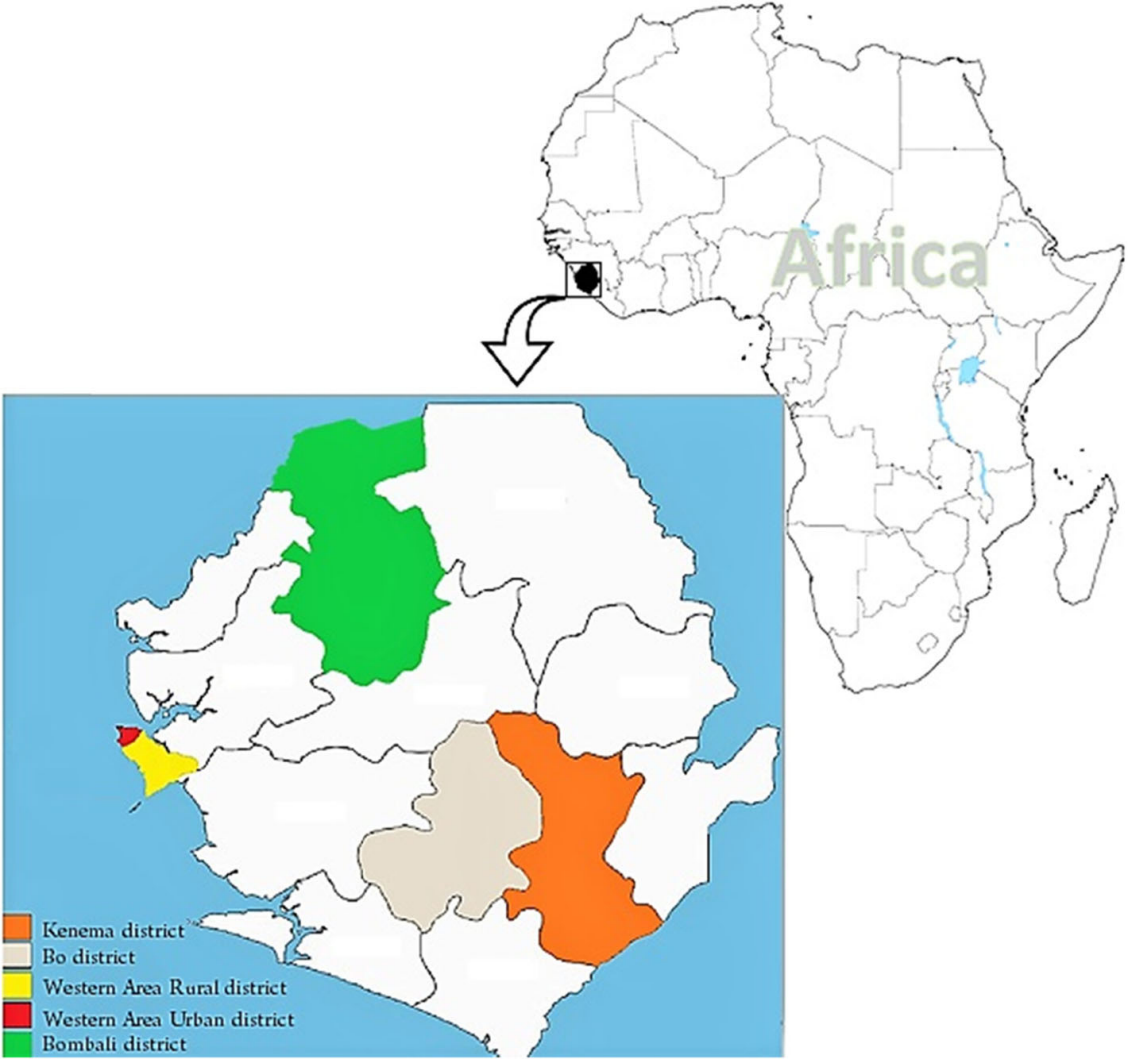
Locations of the five districts (Western area urban, Western area rural, Bombali, Bo and Kenema) in Sierra Leone. (Source: Map created by the authors)

A sample of 351 EVD survivors was determined using a sample size formula for cross-sectional studies (N = ^z2^pq/d^2^). We increased our sample to 400 to make up non-responses. Multistage sampling method was used to recruit participants across the country. Data was collected from the four geographic regions of Sierra Leone (Western Area, Northern province, Southern province and Eastern province). Five districts were purposefully selected to cover all four geographic regions of the country. The location of the five districts in Sierra Leone are shown in Fig. 1. The five districts are western area urban and western area rural districts (both in the Western area), Bo district (Southern province), Kenema district (Eastern province) and Bombali district (Northern province). These five districts were chosen based on the epidemiological profile of the total confirmed Ebola cases and because they are host to the highest number of Ebola survivors in Sierra Leone. We randomly sampled the required number of EVD survivors in all five districts based on proportional representation using the national list of registered Ebola survivors obtained from the Sierra Leone Association of Ebola survivors (SLAES). Survivors who were randomly chosen were invited to participate in the study via telephone.

### Measures

#### Demographics and health related characteristics

The survey instrument measures EVD demographics such age, sex, marital status, educational status, religious affiliation, employment status, financial status, place of residence (urban/rural), geographical region (north, south, east regions and western area) and time (months) since post-discharge. Perceived health status was measured using a five-point Likert scale that ranged from “excellent” to “poor”. EVD survivors were asked if they had been diagnosed with any chronic condition prior to being infected with EVD virus.

#### Ebola-related stigma

The Ebola-related stigma instrument was adapted from the HIV-related stigma for people living with HIV/AIDS (HASI-P). The HASI-P is a validated 33-item scale that measures stigma among HIV/AIDS patients in the past 3 months [43]. This instrument was validated among HIV/AIDS patients in five African countries: Lesotho, Malawi, South Africa, Swaziland and Tanzania. It consists of the following subscales and this includes verbal abuse (eight items, α = 0.886); healthcare neglect (seven items, α = 0.832); social isolation (five items, α = 0.890); fear of contagion (six items, α = 0.795); and workplace stigma (two items, α = 0.758) all of which measures enacted stigma. The final subscale called negative self-perception (five items, α = 0.906) measures internalised stigma [43]. We decided to use HIV/AIDS related stigma scale (HASI-P) because HIV/AIDS patients share similar psychosocial challenges with EVD survivors in terms of social isolation, fear of contagion and family and community stigma and discrimination [44]. In addition, there is widespread misinformation about HIV/AIDS and EVD. For instance, EVD and HIV/AIDS only affects certain groups of people in society (the poor for EVD and promiscuous adults or homosexuals for HIV/AIDS) and the unfounded community fear of being infected with the virus through means that have not being scientifically proven [44]. To adapt to our setting, the HASI-P was reviewed by two experts in sociology and EVD as well as piloted among 10 EVD survivors. Based on their feedback, we decided to remove the two items that measure workplace stigma since the majority of EVD survivors did not have any paid job before or after EVD. We also removed the statement “At the hospital, I was left in soiled bed” from the healthcare neglect subscale since majority of survivors were not admitted at the clinic/hospital. In addition, the wording of some statements were changed to fit the local EVD survivorship context. Further, we decided to assess stigma experienced by EVD survivors since their discharged from Ebola treatment centre instead of the past 3 months, as was the case when the instrument was validated among HIV/AIDS patients [43]. The final adapted HASI-P instrument used in our study is attached as an Additional file 1.

#### Use of traditional and complementary medicine

EVD survivors were asked about their health care utilisation, including whether they have used T&CM treatment (products and practitioners) since their discharge from the ETC. The common T&CM modalities considered in our study were informed by studies undertaken previously in Sierra Leone [31–33, 35, 45–47] and across Africa [30]. We considered T&CM in our study to include biological based therapy (herbal medicine and animal extract), spiritual therapy (prayer/faith healing), alternative medicine systems (Chinese herbal medicine, and acupuncture), and physical therapy/body manipulations (massage therapy, traditional bone setting).

#### Data collection and ethical consideration

Trained data collectors obtained the relevant information from EVD survivors using self-administered or interviewer-administered (for illiterate participants) formats. The University of Technology Sydney Human Research Ethics Committee (UTS-HREC-ETH17–2080) and the Sierra Leone Ethics and Scientific Review Committee granted ethical clearance. A participant information sheet, explaining the purpose and scope of the study, as well as the option to opt out, was given or read (illiterate) to EVD survivors before seeking their consent to participate. Survivors signing or thumb printing the consent form was interpreted as their willingness to participate. Survivors who signed or thumbed printed (for illiterate participants) the consent form were then given the questionnaire to fill or to be interviewed(for illiterate participants).Three hundred and fifty eight EVD survivors consented and completely filled the questionnaire and were included in the data analysis. We collected our data between May and August 2018 and it was done either at the regional office of EVD survivors or their homes or the village courtyard.

### Data analysis

We used IBM SPSS Statistics version 25 to perform all analyses. Each of the 30 stigma items was assigned a score of 0 to 3 (0 = never, 1 = once or twice, 2 = several times and 3 = most of the time). For each participant, we summed the scores and divided by the number of items to get the mean score for each of the factors/subscales. To obtain the overall total stigma mean score, we summed up the mean scores of each of the factors and divided by 30. Stigma was analysed as a binary variable (yes/no). Mean stigma score of zero means that none of the items (experiences) in each of the subscales (internalised stigma, verbal abuse, healthcare neglect, fear of contagion and social isolation) occurred since discharged from the ETC. A mean stigma score greater than zero indicated that at least one of the items in each of the subscales occurred once or twice or several times or most of the time. As a binary variable, mean score of zero was taken as the absence of stigma and greater than zero was taken present of stigma. We employed chi-square and Fischer exact two tailed tests to determine the association between stigma subscales and sociodemographic and health related variables. We conducted a backward stepwise regressions analysis to establish the most parsimonious model that determines the sociodemographic and health related predictors of internal and enacted stigma. We also used backward stepwise regressions analysis to establish the most parsimonious model that predicts whether internal and enacted stigma is an independent predictor of informal healthcare use (T&CM use). To determine the independent association between EVD –related stigma and T&CM use, all of the sociodemographic (age, sex, marital status, religious affiliation, employment status residence etc.) and health related (perceived health status, Duration(years) since discharged from ETC, known chronic disease) variables were taken as potential cofounders and were adjusted for in the regression analysis. Probability less than 0.05 was as statistically significant for all inferential statistical analyses.

## Results

Out of the 400 survivors invited to participate in the study, 377 of them agreed to take part in the study. However, 19 failed to completely fill the questionnaire. Thus, complete data on 358 EVD survivors were analysed. Table 1 gives a summary of EVD survivors’ sociodemographic and health-related characteristics. More than half (*n* = 194, 54.2%) of survivors were within the ages of 18–33 years and close to two-thirds (*n* = 223, 62.3%) were females. Also close to three –fourths (*n* = 262, 73.2%) of survivors perceived their health to be fair/poor.

**Table 1:** Sociodemographic and health-related characteristics of EVD survivors (*N* = 358)

Characteristics	Variables	Total n (%)
Age group	18–33 years	194(54.2)
	34–49 years	134(37.4)
	≥ 50 years	30(8.4)
Sex	Male	135(37.7)
	female	223(62.3)
Educational Status	Non-formal education	147(41.1)
	Primary	44(12.3)
	Secondary	126(35.2)
	Tertiary	41(11.5)
Religious Affiliation	Christianity	92(25.7)
	Islam	266(74.3)
Marital Status	Single	100(27.9)
	Married/Cohabitating	171(47.8)
	Divorced/Separated/widowed	87(24.3)
Financial management	It is impossible/ Difficult all the time	110(30.7)
	difficult some time	238(66.5)
	Not too bad	10(2.8)
Monthly income (Leones)	Less than 500,000	252(70.4)
	500,000 - 1million	94(26.3)
	>1million	12(3.4)
Residential Area	Urban	219(61.2)
	Rural	139(38.8)
Region	Northern region (Bambali district)	120(33.5)
	Southern region (Bo district)	55(15.4)
	Eastern region (Kenema district)	62(17.3)
	Western Area	121(33.8)
Employment status after surviving Ebola	Full time	164(45.8)
	Part time	16(4.5)
	Casual/Temporal	19(5.3)
	Looking for job	100(27.9)
	not in paid work force	59(16.5)
Current perceived health status	Very good/Good	96(26.8)
	Fair/poor	262(73.2)
Duration(years) since discharged from ETC	≤3 years	27(7.5)
	> 3 years	331(92.5)
Known Chronic disease	Yes	46(12.8)
	No	312(87.2)

### Stigma experienced by EVD survivors

Based on the calculated mean scores, EVD survivors reported higher levels of internalised stigma (0.92 ± 0.77) compared to enacted stigma (0.71 ± 0.61). Among the enacted stigma subscale, social isolation (0.96 ± 0.88) and healthcare neglect (0.46 ± 0.53) were the highest and least respectively. We categorised stigma scores into (yes /no) as there was little variability in stigma scores. In general, majority of Ebola survivors endorsed at least one item exploring internalised stigma (*n* = 298, 83.2%) and any of the three subscales measuring enacted stigma (*n* = 333, 93%). Verbal abuse (*n* = 276, 77.1%) and fear of contagion (*n* = 225, 62.8%) were the highest and least reported enacted stigma subscales respectively (see Table 2).

**Table 2:** EVD-related stigma scores experienced by EVD survivors according to the adapted HASI-P stigma instrument

Stigma scales	Mean (SD)	Mean score = zero (None of the items in each of scale was endorsed) n (%)	Mean score ≥ 0(at least one of the items in each scale was endorsed) n (%)
Internalized stigma (negative self-perception subscale)	0.92(0.77)	60(16.8)	298(83.2)
Total enacted stigma (remaining four subscales)	0.71(0.61)	25(7.0)	333(93)
Verbal abuse	0.72(0.76)	82(22.9)	276(77.1)
Healthcare neglect	0.46(0.53)	105(29.3)	253(70.7)
Social isolation	0.96(0.88)	92(25.7)	266(74.3)
Fear of contagion	0.72(0.84)	133(37.2)	225(62.8)

### Association between stigma and sociodemographic and health related variables among Ebola survivors

Table 3 summarises the comparison of internalized and enacted stigma with sociodemographic and health related variables among Ebola survivors. Religious affiliation (*p* = 0.038) and perceived health status (*p* = 0.004) were associated with internalised stigma. None of the sociodemographic and health related variables was associated with enacted stigma. After adjusting for possible cofounders through regression analysis, Ebola survivors who were Christians [AOR = 2.51, 95%CI: 1.15–5.49, *p* = 0.021], who perceived their health to fair/poor were [AOR = 2.58, 95%CI: 1.39–4.77. *p* = 0.003] and who reside in the northern region of Sierra Leone [AOR = 2.80, 95%CI: 1.29–6.07, *p* = 0.009] were more likely to experience internalised stigma (see Table 4). No sociodemographic and health related variables predicted total enacted stigma. Table 5 summarizes the independent association between the individual subscales of the enacted stigma and sociodemographic and health related variables. Ebola survivors who reside in the urban area were more likely [AOR = 2.7, 95%CI: 1.57–4.66, *p* < 0.001] and those who live in the Northern Region of Sierra Leone were less likely [AOR = 0.37, 95%CI: 0.20–0.69 *p* = 0.002] to experience verbal abuse. Survivors residing in the Northern Region compared to those in the Western Area were more likely [AOR = 2.03, 95%CI: 1.13–3.64, *p* = 0.018] to experience healthcare neglect. EVD survivors residing in the Southern [AOR = 3.11, 95%CI: 1.41–6.83, *p* = 0.005] and eastern [AOR = 2.44, 95%CI: 1.19–5.03, *p* = 0.015] regions were more likely to be stigmatised due to fear of contagion than those in the Western area. In addition, EVD survivors in the Eastern region were more likely [AOR = 3.06, 95%CI: 1.34–7.02, *p* = 0.008] to be socially isolated than those in the western area. Unemployed EVD survivors were more likely [AOR = 2.13, 95%CI: 1.26–3.6, *p* = 0.005] to be socially isolated than their employed counterparts.

**Table 3:** Comparison of internalized and enacted stigma with sociodemographic and health related variables among Ebola survivors

Characteristics	Variables	Internalized stigma			Enacted stigma		
		Yes n (%)	No n (%)	*p*-value	Yes n (%)	No n (%)	*p*-value
Age group	18–33 years	158(53.0)	36(60.0)	0.599	179(53.8)	15(60.0)	0.815
	34–49 years	114(38.3)	20(33.3)		125(37.5)	9(36.0)	
	≥ 50 years	26(8.7)	4(6.7)		29(8.7)	1(4.0)	
Sex	Male	111(37.2)	24(40.0)	0.688	129(38.7)	6(24.0)	0.143
	female	187(62.8)	36(60.0)		204(61.3)	19(76.0)	
Educational Status	Non-formal education	127(42.6)	20(33.3)	0.551	135(40.5)	12(48.0)	0.396
	Primary	36(12.1)	8(13.3)		39(11.7)	5(20.0)	
	Secondary	103(34.6)	23(38.3)		120(36.0)	6(24.0)	
	Tertiary	32(10.7)	9(15.0)		39(11.7)	2(8.0)	
Religious Affiliation	Christianity	83(27.9)	9(15.0)	0.038	87(26.1)	5(20.0)	0.499
	Islam	215(72.1)	51(85.0)		246(73.9)	20(80.0)	
Marital Status	Single	83(27.9)	17(28.3)	0.676	94(28.2)	6(24.0)	0.861
	Married/Cohabitating	140(47.0)	31(51.7)		159(47.7)	12(48.0)	
	Divorced/Separated/widowed	75(25.2)	12(20.0)		80(24.0)	7(28.0)	
Financial management	It is impossible/ Difficult all the time	91(30.5)	19(31.7)	0.515	102(30.6)	8(32.0)	0.697
	difficult some time	197(66.1)	41(68.3)		222(66.7)	16(64.0)	
	Not too bad	10(3.4)	0(0.0)		9(2.7)	1(4.0)	
Monthly income (Leones)	Less than 500,000	211(70.8)	41(68.3)		236(70.9)	16(64.0)	0.477
	500,000 - 1million	76(25.5)	18(30.0)	0.699	85(25.5)	9(26.0)	
	>1million	11(3.7)	1(1.7)		12(3.6)	0(0.0)	
Residential Area	Urban	185(62.1)	34(56.7)	0.432	204(61.3)	15(60.0)	0.901
	Rural	113(37.9)	26(43.3)		129(38.7)	10(40.0)	
Region	Northern region (Bambali district)	108(36.2)	12(20.0)	0.084	110(33.0)	10(40.0)	0.619
	Southern region (Bo district)	42(14.1)	13(21.7)		53(15.9)	2(8.0)	
	Eastern region (Kenema district)	51(17.1)	11(18.3)		59(17.7)	3(12.0)	
	Western Area	97(32.6)	24(40.0)		111(33.3)	10(40.0)	
Employment status after surviving Ebola	Full time	134(45.0)	30(50.0)	0.467	149(44.7)	15(60.0)	0.187
	Part time	12(4.0)	4(6.7)		16(4.8)	0(0.0)	
	Casual/Temporal	15(5.0)	4(6.7)		16(4.8)	3(12.0)	
	Looking for job	84(28.2)	16(26.7)		96(28.8)	4(16.0)	
	not in paid work force	53(17.8)	6(10.0)		56(16.8)	3(12.0)	
Current perceived health status	Very good/Good	71(23.8)	25(41.7)		88(26.4)	8(32.0)	
	Fair/poor	227(76.2)	35(58.3)	0.004	245(73.6)	17(68.0)	0.544
Duration(years) since discharged from ETC	≤3 years	24(8.1)	3(5.0)	0.414	26(7.8)	1(4.0)	0.487
	> 3 years	274(91.9)	57(95.0)		307(92.2)	24(96.0)	
Known Chronic disease	Yes	37(12.4)	9(15.0)	0.585	43(12.9)	3(12.0)	0.895
	No	261(87.6)	51(85.0)		290(87.1)	22(88.0)

**Table 4:** Adjusted association between demographic and health-related variables and internalized and enacted stigma

		internalized stigma			Enacted stigma		
Characteristics	Variables	AOR	95% CI	*p*-value	AOR	95% CI	*p*-value
Religion	Islam	1		0.021			
	Christianity	2.51	1.15–5.49				
Perceived health status	Very /good	1		0.003			
	Fair/poor	2.58	1.39–4.77				
Region of country	Western Area	1					
	North region	2.80	1.29–6.07	0.009			
	Southern region	0.85	0.38–1.93	0.700			
	Eastern region	1.14	0.51–2.56	0.749			
Employment status	Employed				2.16	0.88–5.31	0.093
	Unemployed

**Table 5:** Adjusted association between demographic and health-related variables and Individual scales of enacted stigma

		Verbal abuse			Healthcare neglect			Fear of contagion			Social Isolation		
Characteristics	Variables	AOR	95% CI	*p*-value	AOR	95% CI	*p*-value	AOR	95% CI	p-value	AOR	95% CI	*p*-value
Sex	Female				1								
	Male				1.53	0.93–2.51	0.094						
Religion	Islam	1						1			1		
	Christianity	1.95	0.98–3.88	0.056				1.62	0.94–2.80	0.081	1.72	0.92–3.19	0.088
Monthly Income(Leones)	>1million	1											
	< 500,00	0.35	0.04–2.93	0.330									
	500,00-1million	0.16	0.02–1.43	0.101									
Region	Western Area	1			1			1			1		
	North region	0.37	0.20–0.69	0.002	2.03	1.13–3.64	0.018	0.73	0.43–1.23	0.231	1.15	0.65–2.05	0.626
	Southern region	1.19	0.49–2.86	0.701	1.43	0.69–2.96	0.332	3.11	1.41–6.83	0.005	1.75	0.78–3.93	0.174
	Eastern region	1.61	0.65–4.01	0.302	0.81	0.43–1.53	0.506	2.44	1.19–5.03	0.015	3.06	1.34–7.02	0.008
Residence	Rural	1						1					
	Urban	2.7	1.57–4.66	< 0.001				1.50	0.94–2.40	0.090			
Perceived health status	Very good/good							1					
	Fair/poor							1.64	0.98–2.74	0.060			
Employment status	Employed										1		
	Unemployed										2.13	1.26–3.61	0.005

### Association between T&CM use and internalised and enacted stigma

Table 6 presents the independent association between T&CM use and internalised and enacted stigma using backwards stepwise binary logistic regression. EVD survivors who experienced enacted stigma were [AOR = 4.58, 95%CI: 1.51–13.83, *p* = 0.007] more likely to use T&CM. Further analysis of the subscales of the enacted stigma revealed that verbal abuse [AOR = 1.95, 95%CI: 1.09–3.49, *p* = 0.025} and healthcare neglect [AOR = 2.35, 95%CI: 1.37–4.02, *p* = 0.002] were independent predictors of T&CM use among the EVD survivors. Internalised stigma was not found to be a predictor of T&CM use [AOR = 1.93, 95%CI: 0.99–3.75, *p* = 0.054].

**Table 6:** Association between T&CM use and internalized and enacted stigma using backwards stepwise binary logistic regression

Stigma subscale		AOR	95% CI	*p*-value
Internalized stigma (negative self-perception subscale)	No	1		
	Yes	1.93	0.99–3.75	0.054
Enacted stigma (Verbal abuse, Healthcare neglect, Social isolation, Fear of contagion)	No	1		
	Yes	4.58	1.51–13.83	0.007
Verbal abuse	No	1		
	Yes	1.95	1.09–3.49	0.025
Healthcare neglect	No	1		
	Yes	2.35	1.37–4.02	0.002
Social isolation	No	1		
	Yes	1.52	0.86–2.67	0.146
Fear of contagion	No	1		
	Yes	1.53	0.92–2.55	0.102

## Discussion

This is the first nationally representative study to determine the prevalence of stigma, its sociodemographic correlates and association with informal and non-integrated forms of health care such as T&CM use among EVD survivors in Sierra Leone. One key finding from our study is that EVD survivors report high levels of internalised and enacted stigma since discharge from an Ebola treatment centre which is in line with findings from a longitudinal Liberian study that reported high levels of stigma at baseline but lower levels at subsequent follow-up visits [21, 23]. Our finding also resonates with similar short term and smaller sample size cross-sectional studies in Sierra Leone [24, 25, 48], Liberia [20], Guinea [49], and DR Congo [19, 40],which reported that EVD survivors experience several forms of internalised and enacted stigma. Our result identifies higher occurrence of internalised stigma when compared with the occurrence of total enacted stigma experienced by EVD survivors. Our result contrasts to findings reported in a Liberian longitudinal cohort study that employed a different stigma instrument [23] but is in line with a South African study that employed the same stigma tool to measure stigma among HIV/AIDS patients to that employed in our study [50]. The higher frequency of internalised stigma (negative self-perception) among EVD survivors in our study is a cause for concern and warrants further research attention as it can lead to low self-esteem, low self-efficacy, loss of hope for the future and can interfere with life goal achievement [51]. The findings for EVD studies appear to be similar to some other infectious diseases. For example, similar sequelae have been reported among HIV/AIDS patients in Hong Kong [52] and Uganda [53], in which HIV/AIDS patients reported to feel less worthy of themselves, guilt, shame and self-blame for having HIV/AIDS.

The common types of enacted stigma faced by EVD survivors in our study were social isolation, verbal abuse and fear of contagion, all of which are congruent with the common forms of stigma reported by EVD survivors in the wider literature [6]. These findings may be applicable to other emerging infectious disease survivors more generally, as similar forms of stigma from the public and healthcare staff have also been reported among SARS survivors in Hong Kong [54]. Social isolation, verbal abuse and fear of contagion can lead to increased levels of psychological distress, delayed access to medical care, low adherence to medical therapy and reduced quality of life as it has also been reported among HIV/AIDS and mental health patients [55, 56]. Drawing from lessons learnt from HIV/AIDS-related stigma, several EVD survivor-centred and community-driven strategies have been suggested that could contribute to EVD survivors’ recovery and community re-integration. These include community long-term psychosocial counselling for EVD survivors to enhance their coping skills, community education and social support programs for EVD survivors, recruitment and training of trusted opinion leaders that can spread accurate de-stigmatising messages within communities, minimising social isolation and promoting economic empowerment of EVD survivors and EVD affected communities [44, 57].

The mental health impact of surviving Ebola is enormous, and previous studies have reported that psychological distress, anxiety and depression are widespread among Ebola survivors [4, 6]. Although the impact of Ebola –related stigma on mental illness among Ebola survivors is not well understood, stigma induced psychological distress and anxiety have been found to be associated with adverse mental health outcome among HIV/AIDS patients [58]. Since HIV/AIDS and Ebola virus disease share similar stigmatizing attributes [44], it is possible that Ebola –related stigma maybe contributing to the mental health complications among Ebola survivors. Thus, it likely that stigma reduction strategies will help reduce the mental health burden among EVD survivors.

EVD survivors in our study who identified as Christians and reside in the northern region were more likely to experience internalised stigma. The reasons for the high levels of internalised stigma among Christians remain unclear. Going forward, an in-depth ethnography study would be required to explain the high levels of internalised stigma amongst Christians compared to Muslims that was observed in our study. Our study findings also reveal that EVD survivors who perceive their health to be fair/poor are more likely to experience internalised stigma than those who perceive their health to be good. In HIV/AIDS patients, the link between stigma and perceived poor health status is postulated to be because stigma is known to promote poor adherence to treatment, lowers emotional coping and social support networks and reduces access to and usage of health and social services leading to poor health outcomes [26, 38]. The similarity of our findings suggest that similar concerns may be present for EVD survivors. Further studies are needed to explore the link between internalised stigma and religiosity as well as perceived poor health status among EVD survivors in Sierra Leone. Nonetheless, our results have revealed that religiosity, perceived health status and spatial location are potential predictors of internalised stigma among EVD survivors and, that healthcare provider and social workers should consider these characteristics as possible risk factors for internalised stigma among EVD survivors in Sierra Leone.

Further analysis of the enacted stigma subscales revealed verbal abuse was more likely to occur among EVD survivors residing in urban locations when compared to those living in rural areas. Our finding may be explained by the fact that adherence to local bylaws to prevent stigma and discrimination by the community was more prevalent in rural areas compared to urban areas [59]. Also, previously identified urban-rural community differences in knowledge and perception of, and attitude towards, EVD may also explain our finding [60]. Our study also revealed that EVD survivors who are unemployed were more likely to be socially isolated by their communities than their counterparts who were employed. Such a finding maybe explained given that unemployed EVD survivors are likely to be economically and socially dependent on their families and their communities for their wellbeing and, as such are more likely to experience stigma in the form of isolation from their families and communities compared to employed EVD survivors [17, 61].

EVD survivors who experienced healthcare neglect in conventional healthcare settings in our study were more likely to use T&CM. Our finding is not surprising given that healthcare neglect (negative attitude of healthcare providers, long waiting time or being the last person to be seen by the doctor) leads to patient’s dissatisfaction with conventional healthcare - a key driver for T&CM use in the general and sub-health populations in Africa [30]. Thus, it is important for policy makers and health providers to bear in mind that, like other sub-health populations, EVD survivors will likely seek informal healthcare options if they feel neglected by the conventional health system. At policy level, laws are needed that allow EVD survivors to receive appropriate care in a safe environment without being stigmatised or discriminated. In addition, educational interventions to change the negative attitude towards EVD survivors among health providers are required. However, there were also positive attributes identified for T&CM use. The high rate of T&CM use among EVD survivors who experienced enacted stigma (healthcare neglect and verbal abuse) maybe related to the notion that T&CM may serve as a stigma reduction strategy. For instance, T&CM has been used by patients to resist the terminal understanding of HIV/AIDS and believing that HIV/AIDS is chronic rather than a terminal illness [27]. Also, HIV/AIDS patients and SARS survivors have used T&CM practices such as yoga and Tai Chi to create social support groups as people in such settings are less likely to act differently to each other since they share similar health status and experiences [27, 39]. Drawing from the experiences of HIV/AIDS patients and SARS survivors in using T&CM in managing stigma, it is possible that EVD survivors will be using T&CM not only to address their physical health needs but also to as a coping mechanism against the stigma they are experiencing in their communities and at healthcare facilities. As such, there may be a role for integration of some T&CM – where appropriate – to help improve conventional health options for EVD survivors. Going forward, well-designed qualitative research is required to have a deeper understanding of the meanings of T&CM practice in the everyday lives of EVD survivors.

### Limitations

The following limitations must be considered when interpreting our findings. First, our study may suffer from recall bias as we relied entirely on self-reported data. Second, our study employed a cross-sectional design and, therefore we cannot infer causality between independent and outcomes variables. Third, we adapted the HIV/AIDS-related stigma scale (HASI-P) [43] to measure EVD related stigma among EVD survivors, as there is no detailed or validated tool exist for EVD related stigma. We decided to use HIV/AIDS related stigma scale (HASI-P) because HIV/AIDS share similar characteristics with EVD in terms of social isolation, fear of contagion and family and community stigma and discrimination [44]. Finally, our findings are only applicable to EVD survivors in Sierra Leone and may not be representative of EVD survivors in other neighbouring EVD affected countries. Nevertheless, the national nature of this survey represents one of the most representative samples of stigma in EVD survivors.

## Conclusion

The majority of EVD survivors in Sierra Leone experience both internalised and enacted Ebola-related stigma although internalised stigma was the most common in terms of occurrence. To reduce EVD related stigma, and the impacts of such stigma on EVD survivors’ health and wellbeing, EVD outbreak responses should include EVD survivor-centred and community-driven interventions that can help contribute to EVD survivors’ recovery and community re-integration. EVD survivors appear drawn to informal and non-integrated care (T&CM) via both push (i.e. dissatisfaction with conventional care) and pull (i.e. empowerment and social commitments from T&CM). Future research is needed to have a deeper insight of the meanings of T&CM practice in the everyday lives of EVD survivors.

## Supplementary information

**Additional file 1.** Ebola – related stigma Questionnaire.

## Data Availability

Due to confidentiality and privacy concerns, and given the sensitivity surrounding stigma and discrimination among Ebola survivors, our study did not receive approval from the University of Technology Sydney Human Research Ethics Committee and the Sierra Leone Ethics and Scientific Review Committee to publicly share the raw data. Also, Ebola survivors consented to participate in the study on the basis that their data would not be shared with anyone except members of the research team (My Supervisors and I). The raw data informing the findings of this study are stored privately at the University of Technology Sydney data storage platform called Cloudstor. However, upon reasonable request, the anonymised raw data underlying the findings of this study can be made available through the following persons 1. Racheal Laugery, Senior research Ethics officer, University of Technology Sydney Human Research Ethics Committee, University of Technology Sydney, Email: (Racheal.Laugery@uts.edu.au); 2. Edward Foday, Research and Publications Specialist, Sierra Leone Ethics and Scientific Review Committee, Directorate of Policy, Planning and Information, Ministry of Health and Sanitation, Fifth Floor, Youyi Building, East Wing, Freetown, Sierra Leone, Email: efoday@health.gov.sl .

## Abbreviations

ETC: Ebola treatment centre
EVD: Ebola virus disease
T&CM: Traditional and complementary medicine
