# Supplementary material for: An assessment of Ebola-related stigma and its association with informal healthcare utilisation among Ebola survivors in Sierra Leone: a cross-sectional study
Source: BMC Public Health. 2020 Feb 5;20:182. doi: 10.1186/s12889-020-8279-7 (PMC7001224; doi:10.1186/s12889-020-8279-7)
Supplement: Supplementary file 1 — Additional file 1. Ebola – related stigma Questionnaire. [file 12889_2020_8279_MOESM1_ESM.docx]

**Ebola – related stigma Questionnaire**

**Please read through the following list of events that may have happened to you since you were discharged from the Ebola treatment centre. After going through them, please indicate how often it has happened to you because of you being an Ebola survivor.**

|  | **Since your discharged from Ebola treatment centre, how often did the following events happen because of you being an Ebola survivor?** | Never=0 | Once or twice=1 | Several times=2 | Most of the time=3 |
| --- | --- | --- | --- | --- | --- |
| 1 | I was told to use my own eating utensils |  |  |  |  |
| 2 | I was asked not to touch someone’s child. |  |  |  |  |
| 3 | I was made to drink last from a cup |  |  |  |  |
| 4 | Someone mocked me when I passed by |  |  |  |  |
| 5 | I stopped eating with other people |  |  |  |  |
| 6 | At community places, people are afraid to shake hands with me or touch any object I have touched |  |  |  |  |
| 7 | Someone stopped being my friend |  |  |  |  |
| 8 | A friend would not chat with me |  |  |  |  |
| 9 | I was called bad names |  |  |  |  |
| 10 | People sang offensive songs when I passed by |  |  |  |  |
| 11 | I was told that I have no future |  |  |  |  |
| 12 | Someone scolded me |  |  |  |  |
| 13 | I was told that God is punishing me |  |  |  |  |
| 14 | I was made to eat alone |  |  |  |  |
| 15 | Someone insulted me |  |  |  |  |
| 16 | People avoided me |  |  |  |  |
| 17 | People cut down visiting me |  |  |  |  |
| 18 | My family and/or intimate partner ended their relationships with me |  |  |  |  |
| 19 | I was blamed for my status |  |  |  |  |
| **The next set of questions is about your experiences in the hospital or clinic. Since you discharged from Ebola treatment centre, how often did the following events happen because of you being an Ebola survivor?** | | | | | |
| 20 | I was denied health care |  |  |  |  |
| 21 | At the hospital/clinic, the healthcare staff told me all sorts of derogatory statements that made me feel unwelcomed |  |  |  |  |
| 22 | I was quickly dismissed by doctors or nurse while I still need care |  |  |  |  |
| 23 | I was shuttled around instead of being helped by a hospital/clinic staff |  |  |  |  |
| 24 | At the hospital/clinic, I was made to wait until the last |  |  |  |  |
| 25 | In the hospital or clinic, I was totally ignored by healthcare staff |  |  |  |  |
| **These questions are about some of your thoughts or feelings. How often have you thought or felt this way since you discharged from Ebola treatment centre because of you being an Ebola survivor?** | | | | | |
| 26 | I felt that I did not deserve to live |  |  |  |  |
| 27 | I felt less confindent and ashmed to disclose my status to people |  |  |  |  |
| 28 | I felt completely worthless |  |  |  |  |
| 39 | I felt that I brought a lot of trouble to my family and community |  |  |  |  |
| 30 | I felt that I am no longer a person |  |  |  |  |
